# YouTube videos on nutrition and pediatric cancer: scientific reliability and quality

**DOI:** 10.3389/fpubh.2026.1767637

**Published:** 2026-02-27

**Authors:** Saniye Sözlü, Melahat Sedanur Macit-Çelebi, Betül Kocaadam-Bozkurt

**Affiliations:** 1 Department of Nutrition and Dietetics, Faculty of Health Sciences, Tokat Gaziosmanpaşa University, Tokat, Türkiye; 2 Department of Nutrition and Dietetics, Faculty of Health Sciences, Ondokuz Mayıs University, Samsun, Türkiye; 3 Department of Nutrition and Dietetics, Faculty of Health Sciences, Erzurum Technical University, Erzurum, Türkiye

**Keywords:** nutrition, pediatric cancer, quality, reliability, YouTube

## Abstract

**Introduction:**

YouTube is widely used as a source of information on health and nutrition. However, concerns exist regarding the reliability and scientific accuracy of its content. This study aims to evaluate the quality, reliability, and scientific accuracy of YouTube videos related to pediatric cancer and nutrition.

**Methods:**

The reliability of the videos was assessed using the mDISCERN scale, their quality was evaluated with the Global Quality Scale (GQS), and their comprehensiveness was measured using the Nutrition and Pediatric Cancer Scoring System (NPCSS), a scale specifically developed for this study.

**Results:**

Of the analyzed videos, 60% were classified as useful, while 40% were deemed misleading. Highly reliable videos were found to be longer and more comprehensive; however, their viewership rates were significantly lower (*p* < 0.05). The majority (66.7%) of the useful videos were presented by dietitians. Correlation analyses demonstrated strong positive relationships between reliability, quality, and comprehensiveness (*p* < 0.001). Nevertheless, as video quality and reliability increased, viewership rates tended to decrease. The instruments demonstrated an area under the curve ranging between 0.90 and 0.97.

**Discussion:**

YouTube contains limited content on pediatric cancer and nutrition, and only slightly more than half of the available videos are classified as useful. Even these useful videos receive low audience engagement, revealing a gap between content quality and viewer reach. This highlights the need to increase high-quality content and enhance the visibility of reliable information.

## Introduction

Digital platforms have become integral to contemporary life, and there is increasing consultation on their enormous and untapped potential (1). Among digital platforms, YouTube is the leading content community, boasting over one billion daily video views, 30 million active users daily, and 500 h of video submitted every minute. This platform has emerged as a potent instrument for rendering video content worldwide and making it available to a broad audience (2).

Today, YouTube plays an important role in access to information in the health field (3). It is widely used by parents, relatives of patients, and healthcare professionals to learn and raise awareness about serious diseases such as cancer (4). In this context, the quality of educational content provided, especially on the relationship between childhood cancer and nutrition, is very important. Nutrition is a critical factor affecting the disease’s course and patients’ quality of life during cancer treatment. While inadequate or unbalanced nutrition can negatively affect the treatment process, proper nutritional habits can support the fight against the disease (5). Therefore, parents and relatives of patients can access accurate information thanks to educational videos based on scientific foundations (6). Scientific content shared on digital platforms such as YouTube can be an important resource in providing families and health professionals with up-to-date and accurate information. However, choosing reliable and expert-prepared content to access accurate information is of great importance. Nonetheless, as with other digital platforms, YouTube lacks filters or systems to authenticate the veracity of material, presenting a danger of disinformation, particularly for less-informed users (2). In other words, individuals may post videos on YouTube without a reviewer’s approval. Patients could be misled by certain advertisements and posts intended for monetization specifically. Consequently, utilizing these platforms for medical advice or information may result in disseminating inaccurate information. Thus, it is crucial to evaluate the quality and trustworthiness of the data disseminated to millions of people on video-sharing sites such as YouTube (4).

Cancer is one of the leading causes of death around the world (7). Approximately 400,000 children and adolescents (0–19 years old) are diagnosed with cancer year worldwide (8). The rising incidence of pediatric cancer diagnoses continues to position it as a primary cause of non-accidental mortality (9). Investigating the connection between diet and cancer has been a topic for research. Several studies highlight the importance of diet in cancer patients, as well as finding foods that either prevent or promote cancer (10, 11).

Several studies examine the content of cancer-related videos on the digital platform YouTube (4, 12, 13). However, to the best of our knowledge, no study has yet analyzed the content of pediatric cancer and nutrition. This study aimed to identify the most viewed YouTube videos using keywords such as “nutrition in pediatric cancer” and “nutrition in childhood cancer,” evaluate them using internationally recognized scoring systems, and examine the videos’ scientific reliability, quality, and accuracy.

## Methods

This study evaluated the reliability, quality, content, and scope of YouTube (www.youtube.com) videos related to pediatric cancer and nutrition. The terminology frequently used on the subject was analyzed, and nine keywords were determined as “Nutrition in pediatric oncology,” “Nutrition in pediatric cancer,” “Nutrition in childhood cancer, “Diet for pediatric oncology,” “Diet for pediatric cancer,” “Diet for childhood cancer,” “Food for pediatric oncology,” “Food for pediatric cancer”, and “Food for childhood cancer”.

**Figure 1:**
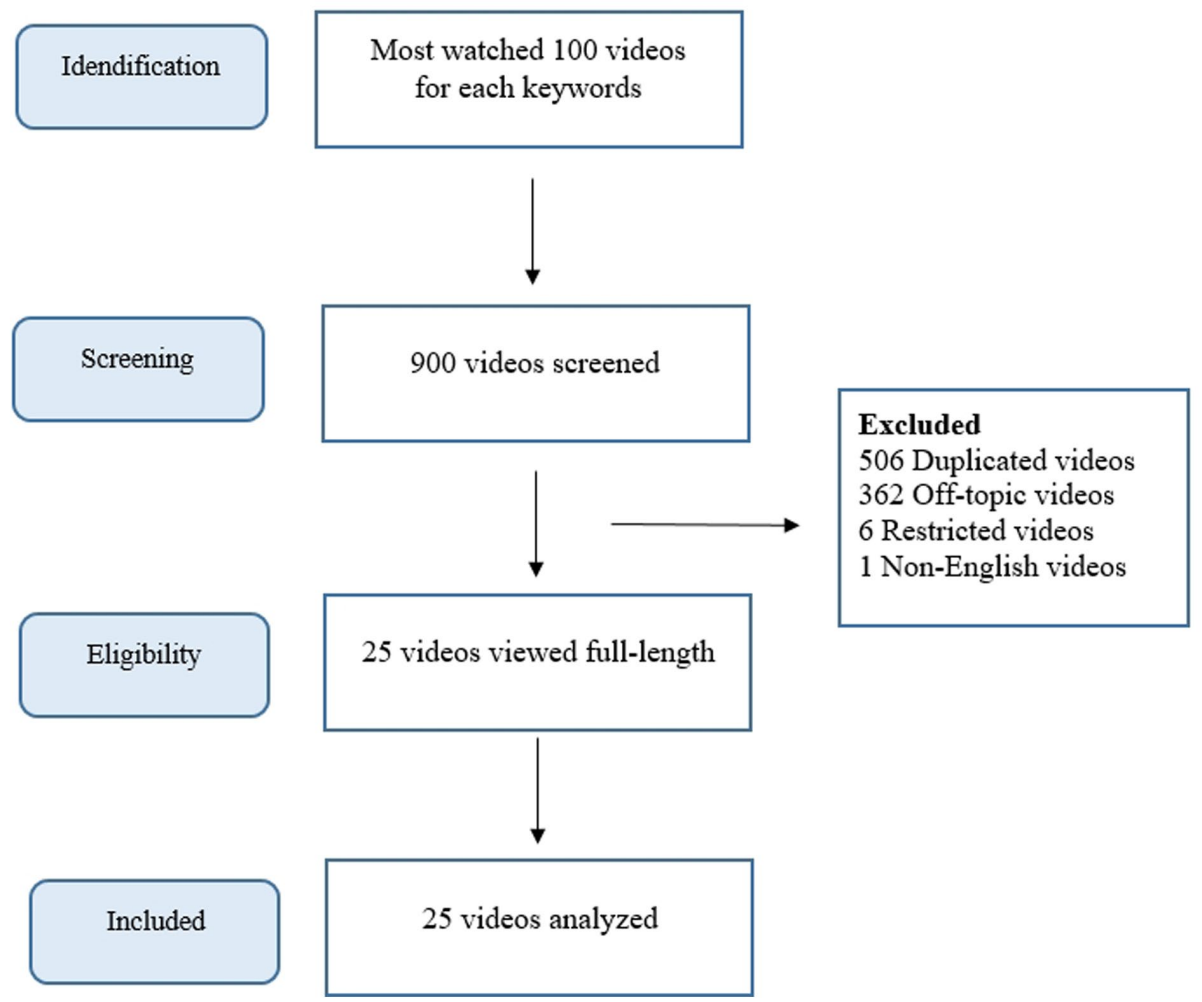
Flowchart of the study.

Before scanning, YouTube search history was deleted, and the search filter was set to the most viewed videos, thus imitating the behavior of ordinary users. URL addresses of 100 videos for each keyword, totaling 900 videos, were recorded for future analysis. This entire scanning and recording process was carried out on May 14, 2024, using the methods used in previous studies, and the analysis process of the videos was started (4, 14). During the video analysis process, duplicate, irrelevant, non-English, audio-only, visual-only, YouTube Short videos (≤60 s), and videos with insufficient audio/visual quality were excluded from the study. After excluding videos that did not meet the criteria, the study was conducted with 25 videos (Figure 1).

### Evaluation of videos

The video metrics, reliability, quality, comprehensiveness, and accuracy of the 25 videos included in the study were evaluated by two academic dietitians (SS, MSMÇ) with more than 10 years of experience in the nutrition and dietetics field. In case of any disagreement, the researchers came together with the participation of the third academic dietitian (BKB) and reached a consensus on the final score.

### Video metrics

The following quantitative metrics were recorded for each video: duration (in seconds), number of views, number of comments, number of subscribers, number of likes and dislikes, and number of days since upload. Additionally, the like rate was calculated as (likes × 100)/(likes + dislikes), view rate (views/days since upload), and video power index (VPI) as (like rate × view rate)/100 (4, 14).

### Assessment of reliability

Video reliability was assessed using the modified DISCERN (mDISCERN) scale. This scale consisted of five questions, and each question was scored as 0: no and 1: yes. In this scale, where the maximum score is five and the minimum score is 0, videos with a score of 3 and above were classified as reliable, and videos with a 2 and below were classified as non-reliable (15) (Table 1).

**Table 1:** Assessment tools of YouTube videos for Nutrition and Pediatric Cancers.

Nutrition and Pediatric Cancer Scoring System (NPCSS)
Assessment of nutritional status
1. Explain nutritional risk screening
2. Explain growth measures for children
3. Describe malnutrition and malnourishment
4. Describe cancer cachexia
Daily nutritional requirements
1. Energy and substrat requirements
2. Clarify nutrition interventions
3. Explains the ability to use supplements
General nutritional recommendations
1. Give information for families (food hygiene and storage, cooking, preparation, and serving etc.)
2. Explain the nutritional recommendations in patients receiving radiotherapy and chemotherapy
Disease related symptoms
1. Mention the disease related nutritional symptoms (such as nausea, vomiting, chewing, swallowing, mucositis etc.)
2. Explain the nutritional recomendations for the symptoms
Reliability (mDISCERN)
1. Are the explanations given in the video clear and understandable?
2. Are useful reference sources given? (publication cited, from valid studies)
3. Is the information in the video balanced and neutral?
4. Are additional sources of information given from which the reviewer can benefit?
5. Does the video evaluate areas that are controversial or uncertain?
Quality (Global quality scale)
1. Poor quality, poor flow, most information missing, not helpful for patients
2. Generally poor, some information given but of limited use to patients
3. Moderate quality, some important information is adequately discussed
4. Good quality good flow, most relevant information is covered, useful for patients
5. Excellent quality and excellent flow, very useful for patients

### Assessment of quality

The educational quality of the videos was assessed using the Global Quality Scale (GQS) tool, which is designed for online resources and consists of five items. Videos were scored on a scale of 1 to 5, with higher scores indicating superior academic quality and excellent information flow (16) (Table 1).

### Assessment of comprehensiveness and accuracy

While scoring methods mDISCERN and GQS assess the quality and reliability of videos uniformly, they may be inadequate for evaluating the comprehension and accuracy of videos on specific subjects, such as pediatric cancer and nutrition. In order to find a solution to this situation, the Nutrition and Pediatric Cancer Scoring System (NPCSS) was developed by researchers after reviewing the literature and taking expert opinions into account (17–19) (Table 1). This scoring system consisted of four subscales and 11 items, and each item was evaluated between 0 and 2 points. In the scoring system, zero indicates that the information is incorrect or incomplete, and one indicates that the information is correct but incompletely mentioned. Two indicates that the information is accurate and detailed. Although the NPCSS scoring system has not yet been validated, disease-specific scoring systems have been widely used in similar studies and have demonstrated their applicability in evaluating video content (20, 21).

### Assessment of usefulness

The videos were evaluated in terms of quality, reliability, comprehensiveness, and accuracy, and these criteria were collectively used to determine their usefulness. Videos containing scientifically accurate and informative content were classified as useful, whereas those deemed unuseful did not necessarily contain incorrect information but often lacked sufficient depth or completeness. The URLs and characteristics of both useful and unuseful videos are presented in Table 2.

**Table 2:** Characteristics and usefulness of YouTube videos on nutrition in pediatric cancer.

No	Video title	URL	Presenter type	Publisher affiliation	Usefulness
1	Paediatric Oncology Series: Nutrition for Children with Cancer	https://www.youtube.com/watch?v=pClH_pvsYxY	Dietitian*	Health care facility/organization	Useful
2	OncoEgypt 2022 - Nutrition 3: Pediatric Oncology	https://www.youtube.com/watch?v=8PzuYfh1Yig	Dietitian*	Health related web sites	Useful
3	Childhood Cancer Symposium: Nutritional Concerns	https://www.youtube.com/watch?v=0D7CXH82s-o	Dietitian	Non-profits	Useful
4	Childhood Cancer Symposium - Nutritional Concerns (1 of 3)	https://www.youtube.com/watch?v=cLDGUhW7PKU	Dietitian	Non-profits	Useful
5	Children with Cancer: Enhancing Nutrition by Katie Nieuwhof and Julia Celestin	https://www.youtube.com/watch?v=uS6tTuAjNbo	Dietitian	Non-profits	Useful
6	Nutrition Tips for Children with Cancer by Ms. Natalie Goh	https://www.youtube.com/watch?v=YLaGN34g0_w	Dietitian	Health related web sites	Useful
7	Childhood Cancer Symposium - Nutritional Concerns (3 of 3)	https://www.youtube.com/watch?v=XU5QdJjteEE	Dietitian	Non-profits	Useful
8	Nutrition for childhood cancer survivors	https://www.youtube.com/watch?v=N38xyr_qTbU	Dietitian	Non-profits	Useful
9	KNH-UoN WEBINAR: Nutrition in Pediatric Oncology	https://www.youtube.com/watch?v=nw7Bm4tBnrY	Dietitian	Health care facility/organization	Useful
10	Education Webinar: Nutrition - During & Beyond Pediatric Cancer Treatment	https://www.youtube.com/watch?v=98OxAa9HyyQ	Dietitian*	Non-profits	Useful
11	The Right nutrition for PEDIATRIC CANCER | TBCY	https://www.youtube.com/watch?v=HsdUWPDJZ2U	Unknown	Non-profits	Useful
12	Eating Well During Cancer Treatment	https://www.youtube.com/watch?v=9ssSJSUpPIM	Unknown	Health care facility/organization	Useful
13	Evaluation of the composition of the pediatric cancer survivor diet as a need for nutrition	https://www.youtube.com/watch?v=XUJRr_45SrQ	Unknown	Personal	Useful
14	“Nutrition for Leukemia Patients: Best Foods to Eat for Managing Symptoms”	https://www.youtube.com/watch?v=1y_pWrpAh-E	Unknown	Health related web sites	Useful
15	Dr. Paul Rogers: The Role of Nutrition in Childhood Cancer	https://www.youtube.com/watch?v=q3UZhMqCoak	Physician	Non-profits	Useful
16	Nutrition in Children | Pediatric Oncologist | Hematologist | Dr. Neema Bhat	https://www.youtube.com/watch?v=mOUfRfek5kg	Physician	Health related web sites	Unuseful
17	How a Nasogastric Tube Can Help with Nutritional Support During Pediatric Cancer Therapy	https://www.youtube.com/watch?v=8PZqdLjy5Tc	Dietitian	Health care facility/organization	Unuseful
18	Nutrition during cancer treatment and recovery - Pediatric Hematology-Oncology at Penn State Health	https://www.youtube.com/watch?v=Flic4YB1ZG8	Physician	Health care facility/organization	Unuseful
19	Special Focus Dialogue - Nutrition and cancer in children, teens and young adults	https://www.youtube.com/watch?v=9FQ0GyXt5Lc	Physician	Non-profits	Unuseful
20	Nutrition During Cancer Treatment | Cincinnati Children’s	https://www.youtube.com/watch?v=faTOz3t06aY	Unknown	Health care facility/organization	Unuseful
21	Dr Inge Huybrechts on nutrition and childhood cancer	https://www.youtube.com/watch?v=2pUPIBdmZJg	Dietitian	Health care facility/organization	Unuseful
22	Live Webchat: Life After Childhood Cancer Treatment	https://www.youtube.com/watch?v=VtMxuAmOj-E&t=53s	Physician	Health care facility/organization	Unuseful
23	5 Easy Tips for Eating Healthy While on Treatment for Children’s Cancer	https://www.youtube.com/watch?v=EOI0eHFVVz4	Patients/ patient’s relative	Non-profits	Unuseful
24	Let us Talk Chemo: Nutrition and eating well	https://www.youtube.com/watch?v=nGir1JaErE0	Dietitian	Health care facility/organization	Unuseful
25	Importance of Nutrition During Your Child’s Cancer Treatment	https://www.youtube.com/watch?v=L7lGh2aKFCk	Unknown	Non-profits	Unuseful

^*^Although the video includes more than one presenter, the nutrition information is presented by a dietitian.

### Compliance with ethical standards

In conducting this study, only publicly available videos on YouTube were evaluated, and no human participants or animals were included. Consequently, as in similar studies in the literature, ethics committee approval was not required.

### Statistical analysis

SPSS 27.0 (SPSS Inc., Chicago, IL, USA) program was used for data analysis. Descriptive statistics were presented as median (IQR), frequencies, and percentage. Shapiro–Wilk test was applied to evaluate the distribution of data. Comparisons between categorical variables were analyzed using the Pearson chi-square and Fisher exact chi-square. The Mann–Whitney U test was applied to examine group differences. Correlations between GQS, mDISCERN, NPCSS, and like ratio–view ratio scores were examined using the Spearman correlation coefficient. Effect sizes were calculated using r for Mann–Whitney U tests and Cramér’s V for categorical variables, with values of 0.10, 0.30, and 0.50 indicating small, moderate, and large effects, respectively (22).

Cohen’s kappa coefficient was used to assess inter-observer agreement for the mDISCERN and NPCSS scores, whereas quadratic weighted Cohen’s kappa was applied for GQS ratings due to the ordinal structure of the scale. Kappa coefficient values were interpreted as follows: values below 0 indicate no agreement, 0.01–0.20 slight agreement, 0.21–0.40 fair agreement, 0.41–0.60 moderate agreement, 0.61–0.80 substantial agreement, and 0.81–1.00 almost perfect agreement (23).

Receiver Operating Characteristic (ROC) curve analysis was used to calculate the cut-off values of mDISCERN, GQS, and VIQI tools to identify qualified YouTube videos according to their usability. The Area Under the Curve (AUC) value must be greater than 0.5 for a measurement tool to be considered significant. Tools with AUC values between 0.50–0.60 are considered unsuccessful, those between 0.60–0.70 are considered weak, those between 0.70–0.80 are considered moderate, those between 0.80–0.90 are considered good, and those between 0.90–1.00 are considered excellent (24). A *p*-value of less than 0.05 was considered statistically significant.

## Results

Inter-observer agreement for the 25 included videos was assessed using Cohen’s kappa. Unweighted kappa was calculated for mDISCERN (*κ* = 0.728, *p* < 0.001) and NPCSS (κ = 0.738, *p* < 0.001), while quadratic weighted kappa was applied for GQS (κ = 0.950, *p* < 0.001).

When the video metrics analyzed, length of each video (second) median (IQR) values were found to be 868 (202–2,977), 120 (61–1,159) for the number of views, 2003 (275–3,884) for the number of subscribers of the accounts uploading the videos, 2 (0.5–9) for the number of likes, 0 (0–1) for the number of dislikes, and 1,095 days (730–3,285) for the duration that the videos were published on the platform. When interaction rates were examined, the like ratio was calculated as 100 (85–100), the view ratio (views/day) was 0.19 (0.07–0.58), and the VPI score was 0.2 (0.09–0.68). In terms of video quality, the GQS was found to be 4 (2–5), with 9 (36%) of the videos being rated as low quality, 2 (8%) as intermediate quality, and 14 (56%) as high quality. Regarding reliability, the mDISCERN score was calculated as 4 (2–5). 16 (64%) of the videos were classified as reliable, and 9 (36%) as non- reliable. Additionally, the NPCSS score was calculated as 10 (6–21) (Table 3).

**Table 3:** Quality, reliability, and content-related characteristics of YouTube videos addressing nutrition in pediatric cancer.

Video features	n (%)	Median (IQR)
Video features		
Length of each video (second)		868 (202–2,977)
Number of views		120 (61–1,159)
Number of subscribers		2003 (275–3,884)
Number of likes		2 (0.5–9)
Number of dislikes		0 (0–1)
Number of days since upload (day)		1,095 (730–3,285)
Like ratio (likes / [likes + dislikes] × 100)		100 (85–100)
View ratio (views/day)		0.19 (0.07–0.58)
Video power index (VPI)		0.2 (0.09–0.68)
GQS Score		4 (2–5)
Low quality videos *n* (%)	9 (36%)	
Intermediate quality videos *n* (%)	2 (8%)	
High quality videos *n* (%)	14 (56%)	
mDISCERN Score		4 (2–5)
Reliable videos *n* (%)	16 (64%)	
Non- reliable videos *n* (%)	9 (36%)	
NPCSS		10 (6–21)

NPCSS, Nutrition and Pediatric Cancer Scoring System; GQS, Global Quality Scale.

**Figure 2:**
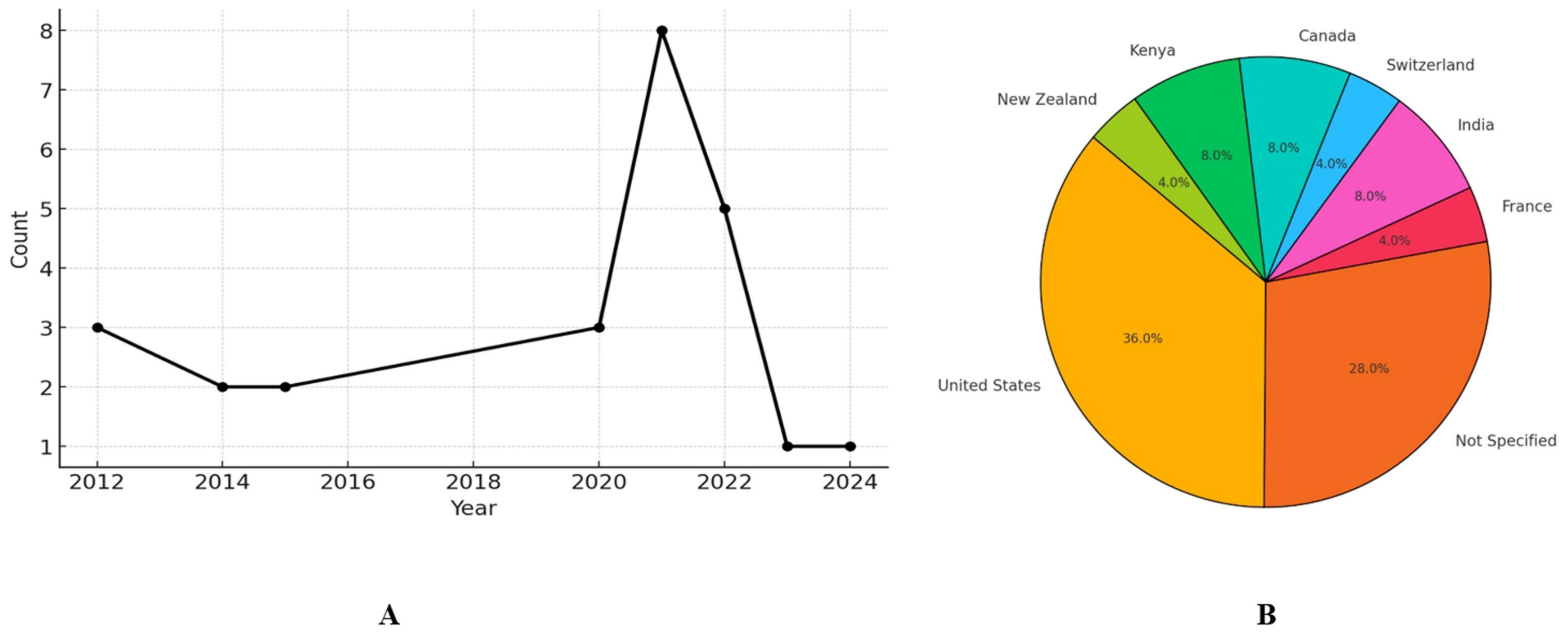
Video counts of each year **(A)**; distribution of videos based on original countries **(B)**.

When the distribution of the number of YouTube videos on pediatric cancer and nutrition by year is examined, it is seen that there were 3 videos in 2012, which constituted 12% of the total videos. The number of videos decreased to 2 (8%) between 2014 and 2015 and remained constant at this level. A gradual increase was observed in 2016–2020, reaching 3 videos (12%) in 2020. A remarkable increase was recorded in 2021, reaching 8 videos, constituting 32% of the total videos, and the peak point was recorded. In 2022, videos decreased to 5 (20%). In 2023–2024, the number of videos decreased significantly to 1 (4%) (Figure 2A).

In the distribution of videos examined by country, most videos originated from the United States, with 9 videos (36.0%) and the most content. There were 7 videos with no specified source, making up 28.0% of the total content. Among other countries, India, Kenya and Canada were represented with 2 videos (8.0%) each. France, Switzerland, and New Zealand were represented with 1 video (4.0%) each (Figure 2B).

Useful videos accounted for 60% of the sample, while 40% were classified as unuseful. Useful videos were significantly longer (*p* = 0.015, r = 0.49) and demonstrated markedly higher educational quality scores, including GQS (*p* < 0.001, r = 0.86), NPCSS (*p* < 0.001, r = 0.84), and mDISCERN (*p* < 0.001, r = 0.76), all indicating large effect sizes. Unuseful videos, on the other hand, were uploaded by channels with a higher number of subscribers (*p* = 0.040, r = 0.41), reflecting a moderate effect size. No significant differences were observed for other quantitative parameters (*p* > 0.05), which showed small to moderate effect sizes. Presenter type also did not differ significantly between useful and unuseful videos (*p* = 0.092), despite a large effect size (r = 0.51). Publisher affiliation showed no significant association with usefulness and had a moderate effect size (*p* = 0.217, r = 0.42) (Table 4).

**Table 4:** Comparative characteristics of useful and unuseful YouTube videos on nutrition and pediatric cancer.

Variables	Useful videos *n* = 15 (60%) Median (IQR)	Unuseful videos *n* = 10 (40%) Median (IQR)	*p*	Effect size (r)
Number of days since upload (day)	1,095 (635–3,285)	1,278 (888–3,361)	0.676	0.08
Length of each video (second)	2,572 (638–3,519)	202 (118–963)	**0.015**	0.49
Number of views	101 (48–371)	1,053 (106–4,359)	0.059	0.38
Number of subscribers	825 (105–2,270)	7,580 (529–88,375)	**0.040**	0.41
Number of likes	1 (0–4)	4.5 (2–28)	0.087	0.34
Number of dislikes	0 (0–2)	0 (0–0)	0.261	0.22
Like ratio	100 (86–100)	100 (67–100)	0.537	0.12
View ratio	0.1 (0.01–0.3)	0.5 (0.1–1.7)	0.059	0.38
VPI	0.1 (0.9–0.3)	0.5 (0.2–1.6)	0.191	0.26
mDISCERN	5 (4–5)	1.5 (1–2)	**<0.001**	0.76
GQS	5 (4–5)	2 (1–2)	**<0.001**	0.86
NPCSS	17 (11–22)	5 (2–7)	**<0.001**	0.84
Presenter type *n* (%)				
Dietitian	10 (66.7%)	3 (30%)	0.092	0.51
Physician	1 (6.7%)	4 (40%)		
Patients/patients’ relatives	–	1 (10%)		
Other health care professions	–	–		
Unknown	4 (26.7%)	2 (20%)		
Publisher affiliation *n* (%)				
Health care facility/organization	3 (20.0%)	6 (60%)	0.217	0.42
Non-profits	8 (53.3%)	3 (30%)		
Personal	1 (6.7%)	–		
Health related web sites	3 (20.0%)	1 (10%)		

NPCSS, Nutrition and Pediatric Cancer Scoring System; GQS, Global Quality Scale; VPI, Video power index. The bold values are indicates significant at *p* < 0.05.

The results of the correlation analysis indicated the presence of high, positive, statistically significant correlation between DISCERN and GQS and NPCS scores (r = 0.863 and r = 0.884; *p* < 0.001, respectively). A similarly very high positive correlation was found between GQS and NPCSS scores (r = 0.943; *p* < 0.001). A low negative but statistically significant correlation was found between the view ratio, mDISCERN, and GQS scores (r = −0.359 and r = −0.404; *p* < 0.05). No significant relationship was found between the like ratio and any parameter (*p* > 0.05) (Table 5).

**Table 5:** Correlation analysis among video characteristics and quality scores of YouTube videos on nutrition and pediatric cancer.

Variables	mDISCERN		GQS		NPCSS		Like ratio		View ratio	
	*r*	*p*	*r*	*p*	*r*	*p*	*r*	*p*	*r*	*p*
mDISCERN	1.000	–	0.863	**<0.001**	0.884	**<0.001**	−0.251	0.299	−0.395	**0.049**
GQS	–	–	1.000	–	0.943	**<0.001**	0.015	0.952	−0.404	**0.045**
NPCSS	–	–	–	–	1.000	–	−0.122	0.618	−0.375	0.065
Like ratio	–	–	–	–	–	–	1.000	–	−0.385	0.103
View ratio	–	–	–	–	–	–	–	–	1.000	–

NPCSS, Nutrition and Pediatric Cancer Scoring System; GQS, Global Quality Scale. The bold values are indicates significant at *p* < 0.05.

**Figure 3:**
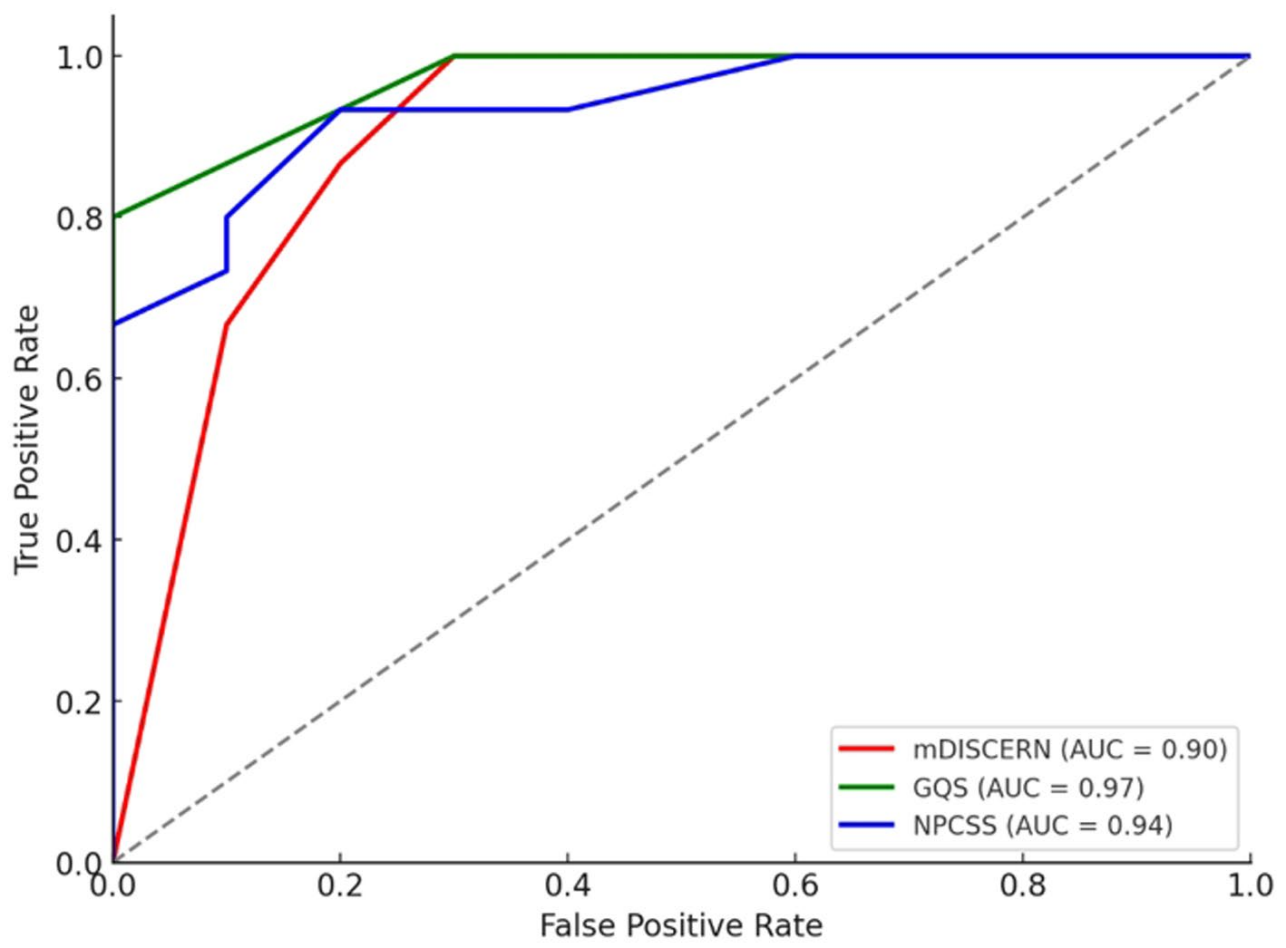
Receiver operating characteristic (ROC) curve for evaluation scores to discriminate highquality YouTube videos based on usefulness.

ROC curve analyses were performed to identify high-quality videos based on the usefulness score. As a result of this analysis, mDISCERN (cut-off point 3.00, AUC 0.90), GQS (cut-off point 4, AUC 0.97) and NPCSS (cut-off point 10.00, AUC 0.94) scores were found to show significant results. All three scores showed above-perfect discrimination ability for high-quality videos (Table 6; Figure 3).

**Table 6:** Receiver operating characteristics (ROC) curve analyses for evaluation scores to discriminate qualifed YouTube videos based on usefulness.

Variables	AUC (95% IC)	Cutt of value	Sensitivity	Specifty	Standart error	*p*
mDISCERN	0.90 (0.84–0.97)	3.00	1.00	0.70	0.033	**<0.001**
GQS	0.97 (0.97–1.02)	4.00	0.80	1.00	0.028	**<0.001**
NPCSS	0.94 (0.88–1.00)	10.00	0.93	0.80	0.031	**<0.001**

NPCSS, Nutrition and Pediatric Cancer Scoring System; GQS, Global Quality Scale; VPI, Video power index. The bold values are indicates significant at *p* < 0.05.

## Discussion

This study evaluated the reliability, quality, comprehensiveness, and accuracy of the most viewed YouTube videos related to pediatric cancer and nutrition. The fact that only 25 of the 900 initially identified videos met the inclusion criteria highlights the extent of information overload and misinformation on this topic. Furthermore, the finding that only 60% of the eligible videos were considered useful indicates that a substantial proportion of the already limited content available on YouTube does not provide meaningful benefit for patients and their families. In addition, the observed decrease in viewing rates as video quality and reliability increased suggests that patients and caregivers may show less interest in scientifically accurate content. Taken together, these findings underscore the challenges patients face in accessing reliable and useful health information on YouTube and emphasize the necessity of the present study.

YouTube is the world’s most visited video-sharing platform and is seen as an important source of information on various topics (4, 25). However, the lack of monitoring mechanisms and the ability of content producers to share videos freely leads to the spread of false and misleading information (25, 26). Today, with the increasing influence of the internet and social media, patients often use YouTube to seek information on health and nutrition (4, 14). YouTube videos on types of cancer and nutrition are also among the most searched content by patients and their relatives (4).

Our study is the first to evaluate pediatric cancer and nutrition videos on YouTube, but numerous studies in the literature examine the overall quality of cancer-related videos. To our knowledge, the only study examining the scientific reliability and quality of cancer and nutrition videos on YouTube is the study conducted by Sütçüoğlu et al. (4). Other studies have evaluated informative videos on specific types of cancer, such as prostate, testicular, lung, bladder, and breast (27–31). These studies show that cancer content on YouTube is highly variable in quality and that a significant portion of it either provides incomplete information or contains false/misleading information. However, the videos that receive the most interaction do not always provide the most accurate information, and sometimes, the opposite occurs, leading to information with a weak scientific basis reaching a 0broad audience (32, 33). This situation poses risks to both patient and general public health.

In the current study, the average length of the videos was approximately 15 min, which is consistent with the view that health-related videos longer than 10 min would increase comprehensiveness (26). However, the views of the videos vary widely (between 3 and 10,570). This shows that while some videos reach large audiences, others fail to attract enough viewers. This finding is similar to the study conducted by Sütçüoğlu and colleagues on cancer and nutrition-related content on YouTube (4). When evaluated in terms of interaction criteria, it is seen that although the like rate is relatively high, the viewing rate (daily views) is relatively low. This finding suggests that viewers generally rate the content positively, but such videos are not popular enough on the YouTube platform. This result is consistent with previous studies that educational and scientific videos generally receive lower interaction (25, 26). Additionally, considering that the average upload time of videos is approximately 3 years, some problems arise in terms of the up-to-dateness of the information. Especially in a rapidly developing field such as pediatric nutrition and cancer, where information must be constantly updated, it can be said that old content may contain false or misleading information. It may have harmful consequences for public health.

This research has examined the distribution of YouTube content on cancer and nutrition between 2012 and 2024. The findings demonstrate that the number of videos on this topic has followed a fluctuating course over the years, with a notable peak especially in 2021. The striking increase recorded in 2021 is particularly noteworthy. This period coincides with increased health consciousness and the search for information on strengthening the immune system due to the effects of the COVID-19 pandemic. Vanderpool et al. have emphasized that nutritional recommendations for cancer patients gained significant popularity during the pandemic period. Additionally, increased screen time and digital content consumption during pandemic restrictions may be among the factors explaining this increase.

According to the analysis of source countries of the examined videos, significant geographical differences have emerged in content production. The United States constitutes the country publishing the most content, accounting for 36% (*n* = 9) of all videos examined. This situation highlights the significant role played by US-based health institutions, researchers, and content creators in the dissemination of information about pediatric cancer nutrition through YouTube. Among other identified countries, three countries—India, Canada, and Kenya—each constituted 8% (*n* = 2) of the total content. The remaining content came from France, Switzerland, and New Zealand, each constituting 4% (*n* = 1) of the total sample. These findings indicate a clear predominance of Western, particularly North American, sources for pediatric cancer nutritional information on YouTube. This geographical distribution pattern may have significant implications for the cultural appropriateness, accessibility, and applicability of nutritional guidance for pediatric cancer patients in different health systems and cultural contexts worldwide.

In the study, when the videos were evaluated in terms of quality, it was found that 56% were of high quality and 36% were of low quality. However, 16 of the 25 videos evaluated (64%) were reliable. This result is often positive. However, many YouTube studies have reported that the videos evaluated are mostly low-quality and have low-reliability scores (26). In a study conducted by Garcia et al. examining YouTube content related to pancreatic cancer, 66.7% of the videos were found to have low reliability (31). Similarly, in a YouTube study by Sütçüoğlu et al. on cancer and nutrition, 61.25% of the videos were found to be unreliabl (4). In contrast, Kahlam et al.’s study of YouTube on colorectal cancer found that most videos were of good quality (34). Thus, YouTube does not provide homogeneous content for health education and that there are significant differences in quality and reliability. It can be said that this situation may cause the spread of false information on cancer issues and pose a potential risk to patient health.

In the current study, the methodologies of previous studies were taken as an example, and the videos examined with the participation of all researchers were divided into two groups useful and misleading. As a result, 60% (n:15) of the 25 videos were classified as useful, and 40% (n:10) were classified as misleading. When the metrics of useful and misleading videos were compared in terms of quality, reliability, comprehensiveness, and accuracy, remarkable results were obtained. First, the median duration of useful videos was approximately 43 min, while the median duration of unuseful videos was approximately 3 min. Sütçüoğlu et al. found in their study that quality videos have a longer duration (4). Second, although there was no significant difference in the number of views, it was observed that channels where unuseful videos were uploaded had more subscribers. This situation can be considered as evidence that YouTube channels that are popular with viewers do not always offer scientifically reliable content. It has been stated in the literature that popular channels can sometimes produce content that contains misleading or incomplete information in order to increase click and view ratio (35). This result highlights the need to carefully evaluate pediatric cancer and nutrition-related videos on YouTube for scientific accuracy.

As a result of evaluating the videos in terms of reliability, quality, and comprehensiveness according to whether they were useful or not, useful videos were found to be significantly higher in quality, more comprehensive, and more reliable. This result indicates that the classification of the videos according to their usefulness was carried out correctly. However, a video being evaluated as unuseful did not always stem from containing incorrect information; in many cases, it resulted from presenting short, superficial, and insufficiently comprehensive content despite including accurate information. Therefore, the absence of a significant difference between useful and unuseful videos in terms of presenter type and publisher affiliation should be interpreted by taking this situation into account. In the literature, it has also been shown that health professionals mostly produce high-quality content in cancer-related studies (4, 36, 37). These findings are consistent with the results of our study. Based on these results, it is important to encourage content produced by health professionals. Additionally, adjusting YouTube algorithms to highlight content approved by reliable institutions will facilitate access to higher-quality information for patients and their relatives.

Correlation analysis conducted in this study revealed strong positive associations among content quality indicators, including mDISCERN, GQS, and NPCSS. This finding indicates that videos with higher quality and reliability tend to provide more comprehensive information and is consistent with the existing literature (4, 30, 36). In addition, a negative relationship was observed between viewing rate and video quality and reliability, suggesting that higher-quality and more reliable videos are relatively less viewed. Similarly, Sütcüoğlu et al. reported that reliable videos related to cancer and nutrition had lower Video Power Index values, indicating reduced audience engagement (4). Previous studies have shown that videos with short and attention-grabbing titles tend to be more popular, whereas more scientific content generally receives lower viewing rates (26, 33). In this context, one possible explanation for the higher view counts of low-quality videos observed in our study is patients’ tendency to seek quick solutions. Additionally, YouTube’s algorithms may further reinforce this pattern by preferentially promoting content that generates higher engagement. This situation represents a significant public health concern, as inaccurate or incomplete information may reach wider audiences and increase the risk of misinformation. Therefore, health-related content on YouTube should be carefully evaluated in terms of scientific accuracy and reliability, and popular videos should not be assumed to be trustworthy. Alongside efforts to improve digital health literacy, it is also crucial that YouTube algorithms prioritize scientifically accurate and reliable content.

## Limitations

This study provides important contributions to pediatric cancer and nutrition but has some limitations. Firstly, due to the dynamic nature of YouTube, videos are constantly updated, new content is added, and popularity metrics change over time. This situation limits the generalizability of the study’s findings and is considered an inevitable limitation. Second, the study focused only on English language videos, which may introduce selection bias. In future studies, evaluating videos in different languages may be useful. Third, how viewers perceive the videos, whether the content is reliable, and how patients or their relatives interpret them have not been examined. Future research should be expanded to include analysis of comments made on videos and investigate how patients are affected by these contents. Fourth, as reported in previous studies, scoring systems used for video content evaluation, including the GQS, mDISCERN, and NPCSS, are inherently subjective and have not always undergone rigorous validation processes. Therefore, the development of more comprehensive and reliable assessment tools is warranted in future research. In addition, the small number of included videos limits the generalizability of the findings and the statistical power of the analyses. However, the fact that only a limited number of videos met the inclusion criteria despite the extensive content available on YouTube highlights both the rigor and the relevance of this study.

## Conclusion

This finding indicates that pediatric cancer and nutrition is an insufficiently represented topic on the YouTube platform. Although many of the reviewed videos included high-quality and reliable information, only a portion of them met the criteria to be classified as useful. Furthermore, it was found that most of the useful videos were presented by dietitians. Another finding of the study was that viewer tendencies did not align with scientific accuracy and content quality. In sensitive topics such as pediatric cancer and nutrition, patients and their relatives should prioritize videos presented by healthcare professionals rather than relying on popular content. In this context, increasing the visibility of trustworthy health content on YouTube and supporting educational initiatives aimed at improving digital health literacy may be beneficial.

## Data Availability

The raw data supporting the conclusions of this article will be made available by the authors, without undue reservation.
